# Management of Muscle-Invasive Bladder Urothelial Carcinoma in a South Asian Adult Man: Utility of Adaptive Radiotherapy in a Resource-Limited Country

**DOI:** 10.7759/cureus.89460

**Published:** 2025-08-06

**Authors:** Sadia Sadiq, Arham Amir Khawaja, Haseeb Mehmood Qadri

**Affiliations:** 1 Radiation Oncology, Institute of Nuclear Medicine and Oncology (INMOL) Atomic Energy Cancer Hospital, Lahore, PAK; 2 General Surgery and Surgical Oncology, Shaikh Zayed Medical Complex, Lahore, PAK; 3 General Surgery, Lahore General Hospital, Lahore, PAK; 4 Neurological Surgery, Punjab Institute of Neurosciences, Lahore, PAK

**Keywords:** adaptive radiotherapy, bladder cancer, limited resources, low- to middle-income countries, oncology, pakistan, planned treatment volume, urinary bladder

## Abstract

Urinary bladder cancer contributes significantly to the global cancer burden and is more prevalent in the developed world. We present the case of a 54-year-old male smoker who underwent transurethral resection of bladder tumor and consequent trimodality therapy (induction chemotherapy followed by concomitant chemo-radiotherapy). His disease was staged at cT3N0M0. Cone beam computed tomography (CBCT) and plan-of-the-day adaptive radiotherapy (ART) were utilized to treat muscle-invasive bladder urothelial carcinoma (MIBC). Instead of the usual planning treatment volume (PTV) of 2 cm, the disease-free state was achieved with a small PTV of 5 mm in 18 fractions and a large PTV of 1.5 cm in two fractions only. The volume of bowel saved with this strategy was 180 cc. The patient tolerated the treatment well with no side effects and is living a healthy life till date. CBCT-guided ART with plan-of-the-day approach is a precise and effective approach for the management of MIBC within limited resources.

## Introduction

Bladder urothelial carcinoma is considered to be the most common malignancy in the genitourinary tract in human beings, with a higher incidence in developed countries compared to third-world countries. The risk factors of bladder urothelial carcinoma are multiple; however, cigarette smoking, metabolic syndrome, alcohol intake, and occupational exposure are the most commonly linked factors. Radical cystectomy and bladder preservation therapy are the available treatment options depending upon the stage of the disease and the patient's preference [1]. Trimodality therapy comprises maximal resection of the bladder tumor and concomitant chemoradiation. Conventionally fixed box technique and 3D-conformal radiotherapy have been used to guide radiotherapy treatment regimens for bladder urothelial carcinoma. Especially in the last two decades, radiotherapy techniques have evolved to reduce toxicity to organs-at-risk (OARs) and increase precision and accuracy in target coverage [2]. Volumetric modulated arc therapy (VMAT) is a refined version of intensity-modulated radiation therapy (IMRT) [3]. Adaptive radiotherapy (ART) is an emerging technique to optimize, guide, and revise treatment plans during the course of patient management [4].

In spite of global ART uptake, data are lacking from resource-limited regions such as Punjab, Pakistan. Khokhar et al. document the access of radiotherapy treatment to 21.4% patients only [5]. Sociocultural barriers and lack of skilled professionals are the most important contributors to healthcare disparity in the field of radiation oncology [6]. We are reporting this case to emphasize the significance and effectiveness of cone beam computed tomography (CBCT)-directed ART in the management of muscle-invasive bladder cancer in resource-limited settings.

## Case presentation

A 54-year-old, smoker, non-hypertensive, and non-diabetic man presented to a public sector hospital with gross hematuria. There was no history of urgency, frequency, or dysuria. The general physical examination was unremarkable, and there was no significant locoregional lymphadenopathy. The cystoscopy revealed growth at the lateral wall of the urinary bladder, and subsequently, the patient underwent transurethral resection of bladder tumor (TURBT) in June 2023. Histopathology was suggestive of high-grade papillary urothelial carcinoma involving the deep muscular layer of the urinary bladder. His contrast-enhanced computed tomography (CECT) and magnetic resonance imaging (MRI) of the pelvis and perineum showed a clinical T3N0M0 disease with a 2 cm residual tumor and peri-vesical fat infiltration. Following a multidisciplinary team meeting at the parent hospital, the patient was referred to the Institute of Nuclear Medicine and Oncology (INMOL) Atomic Energy Cancer Hospital for bladder preservation trimodality therapy (induction chemotherapy followed by concomitant chemo-radiotherapy (CCRT)).

The patient was given three cycles of induction chemotherapy comprising intravenous gemcitabine (1000 mg/m^2^) and intravenous cisplatin (80 mg/m^2^) on day 1 of each cycle and gemcitabine alone (1000 mg/m^2^) on day 8 of each cycle, repeated every three weeks. He had no noticeable side effects during this period, and his hematological investigations were within normal limits.

The patient was planned for CCRT, 55 Gy in 20 fractions, five days a week for four weeks at the rate of 2.75 Gy per fraction using VMAT along with weekly concurrent intravenous cisplatin (40 mg/m^2^).

**Figure 1:**
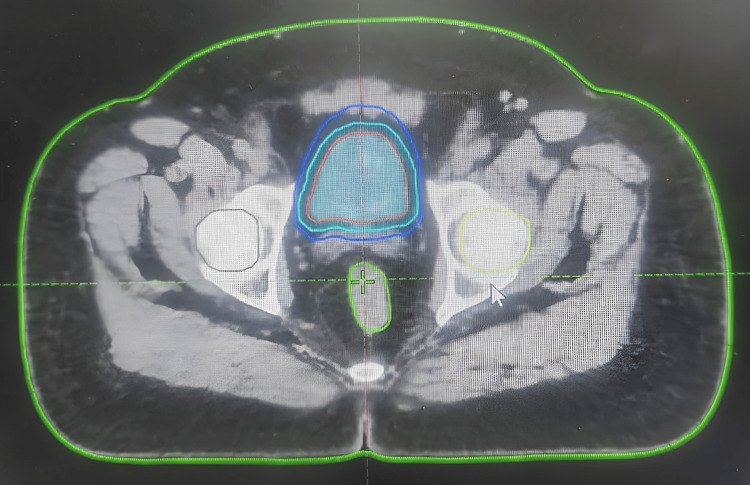
Planning computed tomogram with CTV, small PTV, and large PTV and OARs red: CTV; cyan blue: small PTV; dark blue: large PTV; yellow: left femoral head; brown: right femoral head; green: rectum CTV: clinical target volume; PTV: planning target volume; OAR: organs-at-risk (rectum and femoral heads)

While planning for CCRT, as the urinary bladder is a structure subjected to physiological variability, we planned to use image guidance to treat this patient with ART with plan-of-the-day approach. After informed consent, positioning, and CT simulation with an empty bladder, the patient's data was transferred to the treatment planning system (TPS). The whole of the urinary bladder was part of the high-risk clinical target volume (HCTV). The usual practice of planning treatment volume (PTV) of 2 cm at the author's institution was modified in the index case; two PTVs were made: a small PTV with a 5 mm margin and a large PTV with a 1.5 cm margin all around (Figure 1).

**Figure 2:**
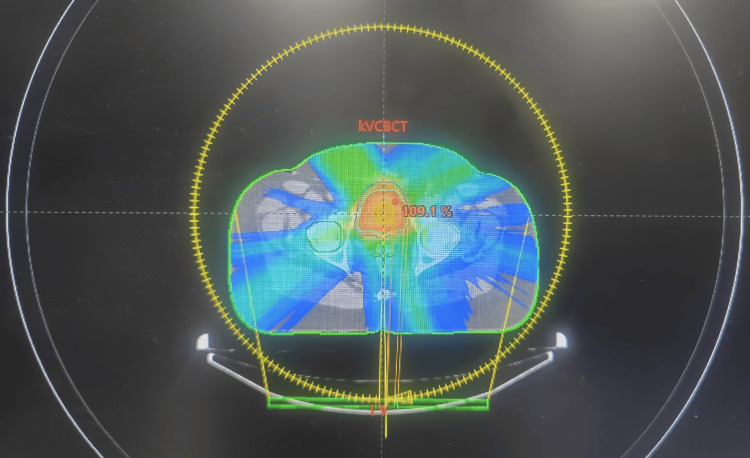
VMAT plan showing the dose color wash VMAT: volumetric modulated arc therapy

After contouring, a library of plans was generated by our physicist, one for each PTV (Figure 2). Prophylactic pelvic nodal radiation was not planned. Two RT plans were calculated, one for each PTV. After the plan evaluation, the patient was asked to come with an empty bladder for the treatment. On the day of treatment, CBCT confirmed bladder volume, and when it was feasible, a small PTV plan was executed. On other days, large PTV plans were used.

**Figure 3:**
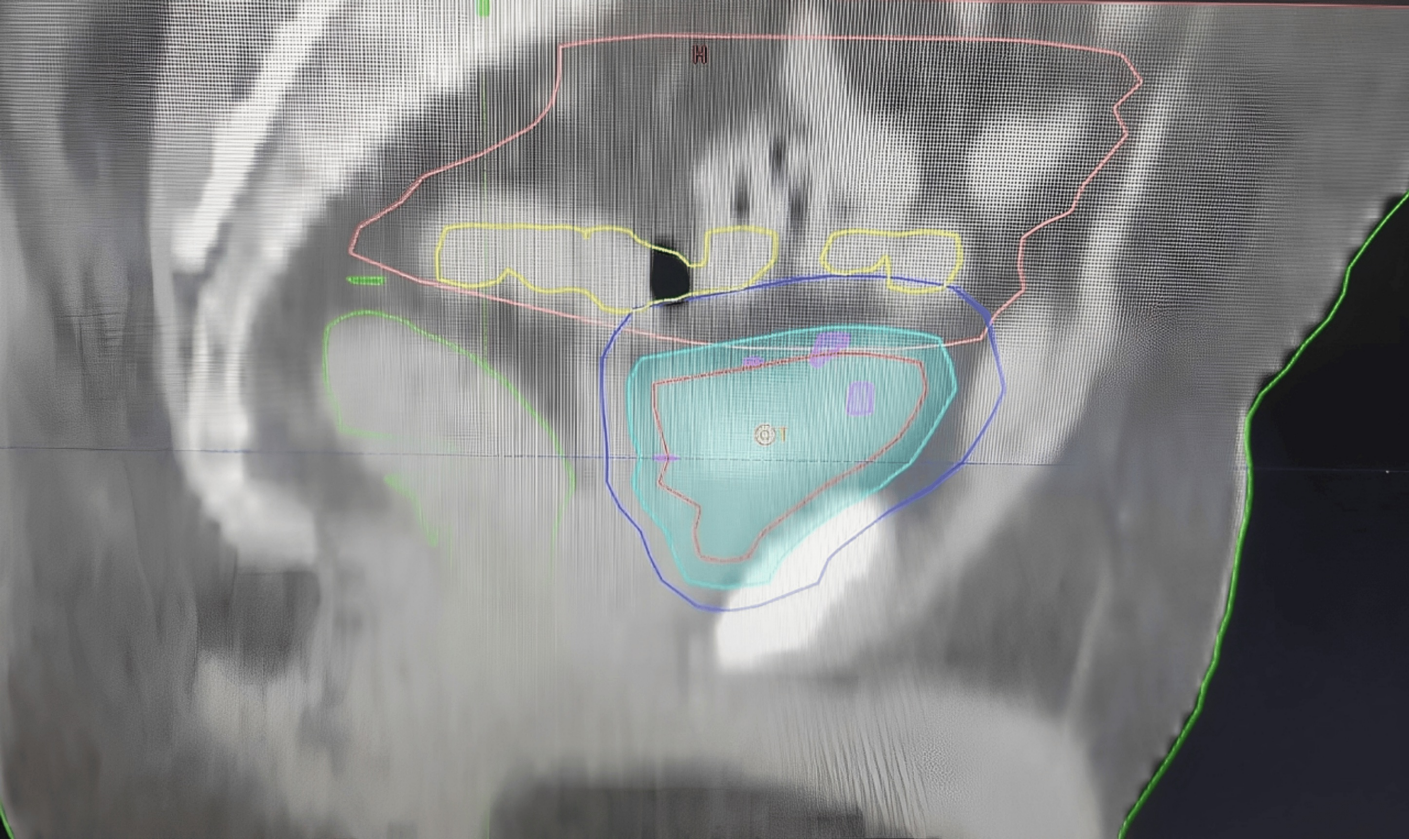
Planning CT sagittal view with an empty bladder, CTV, small PTV, and large PTV red: whole bladder; cyan blue: CTV; dark blue: PTV; pink: bowel bag; yellow: colon; green: rectum CT: computed tomography; CTV: clinical target volume; PTV: planning target volume

**Figure 4:**
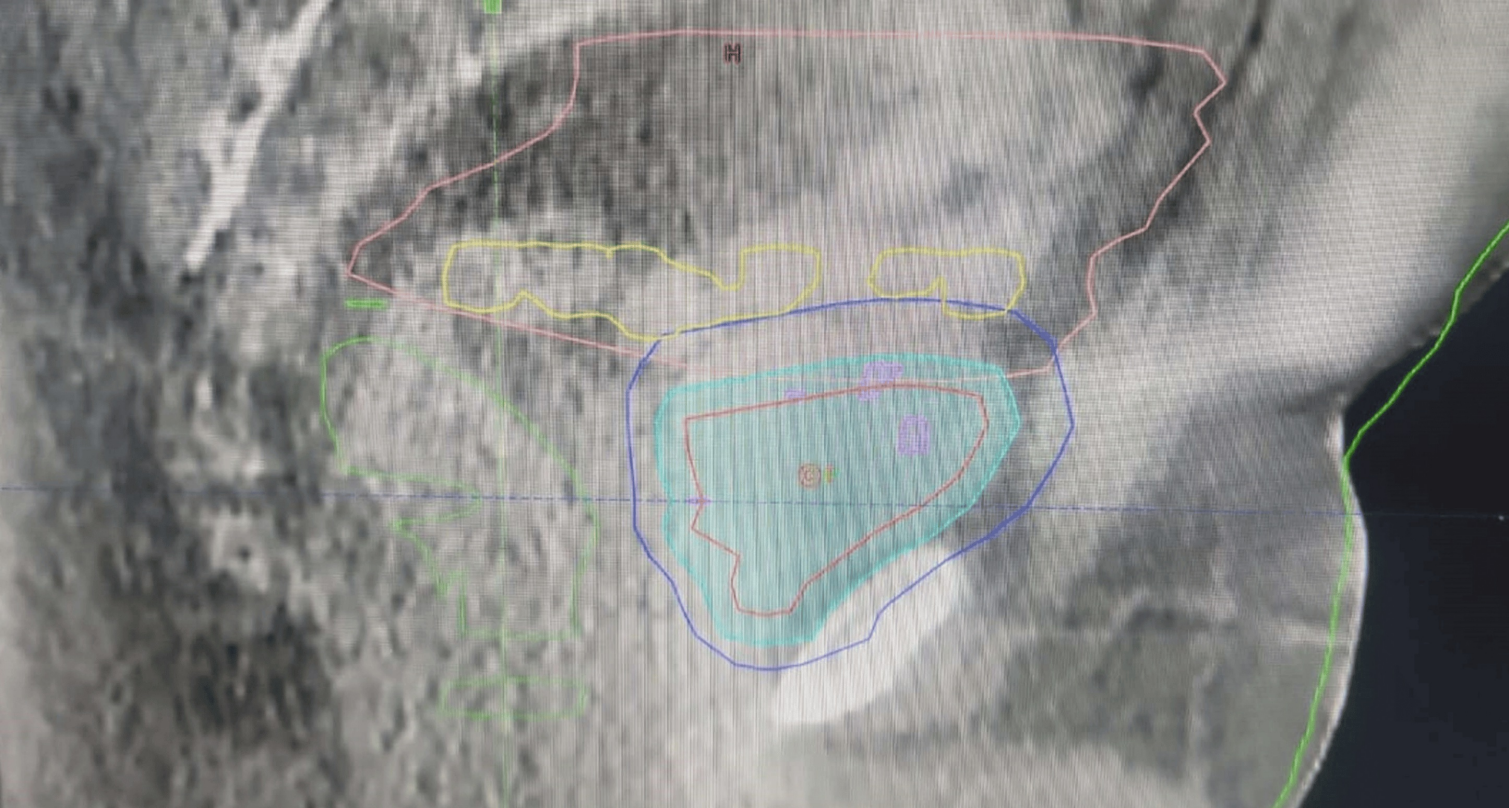
CBCT showing bladder shift beyond small PTV and within large PTV red: whole bladder; cyan blue: CTV; dark blue: PTV; pink: bowel bag; yellow: colon; green: rectum CBCT: cone beam computed tomography; PTV: planning treatment volume

The patient's data was transferred to our treatment machine, Halcyon. The patient was instructed to come for treatment with an empty bladder. On the day of treatment, after getting CBCT, the most suitable plan was selected according to bladder filling on that particular day (Figures 3-4).

The volume of bowel receiving 50 Gy (V50) was 9 cc with the small PTV plan, while it was 19 cc with the large PTV plan. The patient was treated using the small PTV plan in 18 fractions, while the large PTV was used in only two fractions. Consequently, the volume of bowel saved with this plan-of-the-day ART technique was 180 cc during a total of 20 fractions. The patient tolerated the treatment well with no grade 2 or higher acute side effects. It is to highlight that we planned all offline sessions of ART for this patient, as bladder motion changes slowly over time. 

After the treatment, the patient was put on a three-monthly follow-up with cystoscopy and ultrasound and a six-monthly CECT. The first follow-up showed a complete response with no residual tumor or thickening. There were no treatment-related significant adverse effects, which included grade 1 diarrhea during the third and fourth weeks of RT. He was followed for two years post-treatment. 

## Discussion

Offline ART, still in its infancy, holds a promising alternative to standard regimens in the targeted management of bladder urothelial carcinoma and, at the same time, in minimizing the side effects. Online ART is best when motion is rapid and unpredictable (e.g., respiratory motion), allowing real-time adjustment for high precision. Our study reports a middle-aged South Asian man with stage III bladder urothelial carcinoma with no definitive family history of bladder malignancy, but a 20-pack-year history of tobacco smoking. Davis et al. reported a node-positive bladder urothelial carcinoma individual (78 years), incorporating ART to minimize the gastrointestinal side effects [7]. Certain retrospective studies and audits signify a moderate number of patients being undertaken for variations in online ART with dosimetric benefits [8-10]. After an extensive literature search, we conclude there is a lack of adequate comparison between races, descents, and variation in responses, which could be drawn from reported studies. While all reports show effective responses from online ART, the long-term benefits and effects on different target populations versus offline ART are under debate.

In our case, the patient was subjected to a multi-modal approach with initial chemotherapy (consisting of both gemcitabine and cisplatin) followed by ART augmented by cisplatin weekly. A systematic review demonstrated the use of cisplatin-based chemotherapy in improving outcomes for muscle-invasive bladder urothelial carcinoma [11]. However, no study demonstrates the role of ART in combination with chemotherapy. Our case did not develop significant complications related to the combination regimen. Furthermore, the three- and six-monthly follow-ups showed no residual disease or recurrence.

In our case, we used a standard dose of ART (55 Gy in 20 fractions). Huddart et al., in the phase 2 RAIDER randomised controlled trial, compared the use of dose-escalated ART with whole bladder radiotherapy and standard-dose radiotherapy [12]. The results of the study pointed to a low toxicity profile despite escalated doses of radiotherapy. A series of 16 consecutive patients underwent online ART for two or more fractions (total dose of 64 Gy). To summarize, in total, 297/512 fractions were delivered as online ART, with the remaining fractions non-ART (due to variation in staff expertise). The use of a standard regimen of doses of radiotherapy is yet to be developed according to the natural toxicity of doses being delivered to the specific target population [12].

The use of image guidance with CBCT in ART with the VMAT technique helped us select daily suitable plans for the patient based on day-specific geometrical variations of bladder anatomy. Kochan et al. identified that standard radiation treatment for muscle-invasive bladder urothelial carcinoma acquired the significant problem of great variability in mean bladder volumes (154.17±129.38 cm^3^), which resulted in many fractions falling out of the clinical target volume (CTV) [13]. This problem is significantly reduced in our case using ART.

ART was delivered to our patient with a bladder empty protocol. A retrospective audit compared both bladder filling and bladder emptying protocols, providing evidence of the bladder emptying protocol being superior for online ART. Therefore, bladder emptying protocol results in a better CTV, and adequate PTV coverage is achieved with lower ART doses [10].

Our case advocated the use of the plan-of-the-day approach for the optimization of IMRT under CBCT guidance. A case series published from France supports this approach and demonstrates a reduction in irradiated healthy tissue volume and similar bladder coverage with acceptable levels of toxicity [14]. Another study used artificial intelligence to deliver CBCT-guided ART with full optimization to the anatomy of the day [8]. It is important to highlight that a tailored approach is necessary according to the patient's disease burden and anatomical landmarks. Owing to the lack of automated replanning software, we made the best use of the plan-of-the-day approach for our patient.

## Conclusions

CBCT-guided ART enables precise, individualized radiotherapy for muscle-invasive bladder tumors, significantly improving treatment accuracy while minimizing radiation exposure to surrounding healthy tissues. The plan-of-the-day approach effectively mitigates the impact of bladder volume variations, enhancing therapeutic outcomes. These findings underscore the potential of ART as a viable alternative to more invasive interventions, such as radical cystectomy and standard radiation therapy, emphasizing its role in optimizing bladder urothelial carcinoma management.
